# Fetal Magnetic Resonance Imaging of Malformations Associated with Heterotaxy

**DOI:** 10.7759/cureus.269

**Published:** 2015-05-21

**Authors:** Rohit Loomba, Parinda H Shah, Robert H Anderson

**Affiliations:** 1 Cardiology Dept., Children's Hospital of Wisconsin; 2 Department of Radiology, Advocate Illinois Masonic Medical Center; 3 Institute of Genetic Medicine, Newcastle University

**Keywords:** heterotaxy, isomerism, magnetic resonance imaging, mri, double outlet right ventricle, atrioventricular septal defect, malrotation

## Abstract

Magnetic resonance imaging (MRI) is increasingly used as an investigation during fetal life, particularly for assessment of intracranial masses, congenital diaphragmatic hernia, myelomeningocele, and abdominal masses. As the number of scans increases, so is the variety of congenital malformations being recognized. It is axiomatic that interpretation of the findings is enhanced when attention is paid to the likely findings in the setting of known syndromes, this information then dictating the need for additional acquisition of images. One such syndrome is so-called “visceral heterotaxy”, in which there is typically an isomeric, rather than a lateralized, arrangement of the thoracic and abdominal organs. Typically associated with complex congenital cardiac malformations, heterotaxy can also involve the central nervous system, and produce pulmonary, gastrointestinal, immunologic, and genitourinary malformations. In this review, we discuss how these findings can be demonstrated using fetal MRI.

## Introduction and background

Magnetic resonance imaging (MRI) was initially used during fetal life in the early 1980s. Since then, it has become more widely used, although its applications are still limited [1-5]. While ultrasound is still the most commonly used modality to image the fetus, MRI has started to become used for evaluation of the central nervous system, bronchopulmonary, and abdominal malformations [6-14]. Cranial, pulmonary, and abdominal masses can also be evaluated successfully using fetal MRI [15-18]. More recently, cardiac malformations have increasingly been identified using fetal MRI [19-20]. The technique offers improved delineation of anatomy when compared to echocardiography, providing multiple viewing planes, and is not limited by maternal obesity, oligohydramnios, or the fetal lie [21].

So-called “heterotaxy” is a syndrome characterized by abnormal lateralization of the thoracic and abdominal organs, which are arranged in fashions other than the expected arrangement [22]. Cardiovascular, pulmonary, central nervous system, gastrointestinal, and immunologic malformations can all be present in the setting of heterotaxy, and often present in specific combinations. Clinically, the syndrome can be anticipated when there is an intracardiac lesion or caval venous abnormality in the presence of any one of the following: right-sided heart, abnormal arrangement of the abdominal organs, splenic abnormalities, bronchial isomerism, or intestinal malrotation. Historically, the syndrome was segregated on the basis of the splenic anatomy into asplenia and polysplenia. This is now recognized as being less than ideal since splenic anatomy is not the best discriminator of the two subsets of heterotaxy [23]. Despite initial skepticism, it is now well-recognized that isomerism of the thoracic organs, involving the atrial appendages of the heart, provided better segregation. Right isomerism, for example, is typically associated with complex congenital cardiac malformations, such as complete unbalanced atrioventricular septal defects, along with an absence of the spleen, and intestinal malrotation. Left isomerism, in contrast, is more frequently associated with interruption of the inferior caval vein, and multiple spleens, but less severe intracardiac lesions [24-26]. Heterotaxy may be better described as right or left isomerism as this better conveys the arrangement of the visceral organs.

Many of the malformations associated with heterotaxy can now be detected by use of fetal MRI. Recognition of the syndrome is important as an indicator to the need for further evaluation of other organ systems, as well as the likely presence of intracardiac lesions. It also points to the likelihood of impaired splenic function, even when there is a normally located solitary spleen, or multiple spleens [27-29]. Survival, particularly in those patients with right isomerism and functionally univentricular hearts, is also different from those with left isomerism, although the latter feature is known to be the harbinger of complete heart block and fetal hydrops [30]. In this review, we discuss how fetal MRI enhances the diagnosis and evaluation of the findings associated with heterotaxy.  

## Review

### Central nervous system malformations

**Figure 1:**
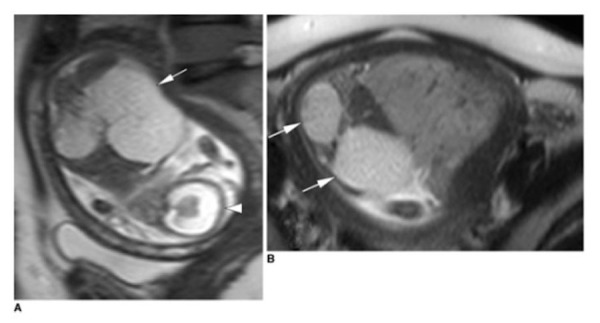
Holoprosencephaly and cortical atrophy Panel A is a sagittal half-fourier acquisition single-shot turbo spin-echo (HASTE) image demonstrating holoprosencephaly and cortical atrophy hydrocephaly. The falx cerebri and interhemispheric fissure are also absent (arrowhead). The white arrow points to an enlarged kidney. Panel B demonstrates an axial HASTE image demonstrating enlarged kidneys with multiple cysts (white arrows). Image reprinted without changes from Koplay, et al. under the creative commons license.

**Figure 2:**
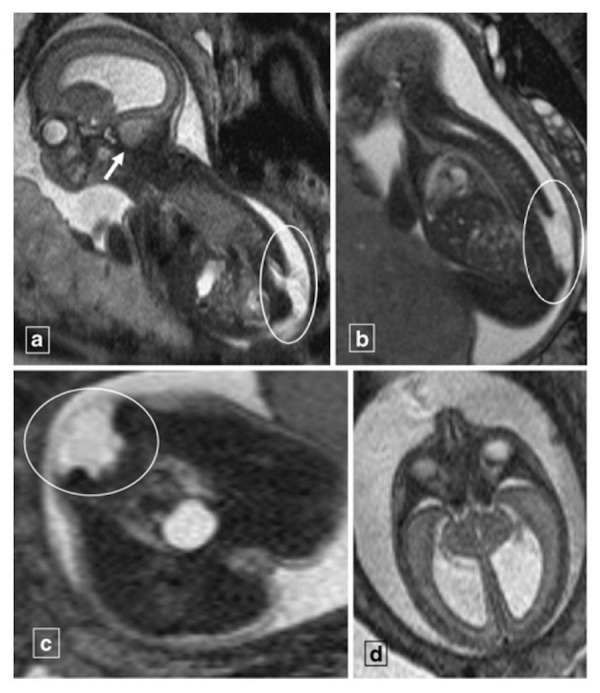
Neural tube defect with tonsillar herniation Panel A is a T2-weighted sagittal image of a 23-week gestational age fetus demonstrating a lumbosacral neural tube defect (encircled) with cerebellar tonsillar herniation (arrow). Panel B demonstrates is a T2-weighted sagittal image demonstrating a myelomeningocele (encircled) from L2 to the end of the sacrum. Panel C demonstrates the myelomeningocele (encircled) in the axial plane. Panel D is a T2-weighted axial image demonstrating hydrocephalus. The findings are consistent with Chiari II malformation. Image reprinted without changes from Nemec, et al. under the creative commons license.

**Figure 3:**
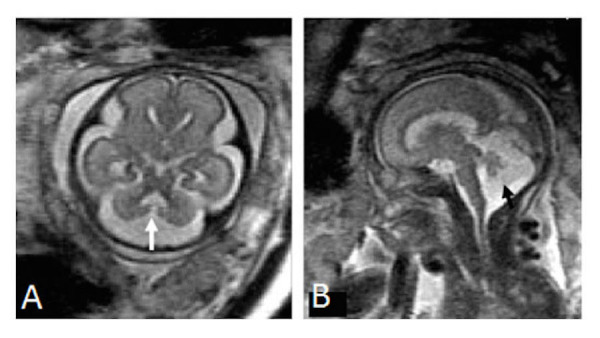
Dandy-Walker malformation Panel A is an axial image demonstrating a direct communication between the fourth ventricle and cisterna magna while Panel B is a sagittal image demonstrating an enlarged posterior fossa, findings consistent with Dandy-Walker malformation. Image reprinted without changes from Sohn, et al. under the creative commons license.

**Figure 4:**
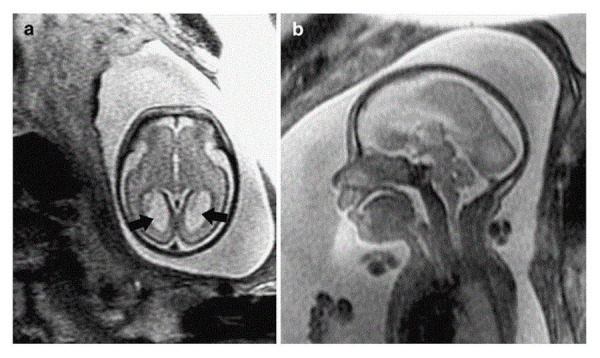
Colpocephaly Panel A is an axial T2-weighted single shot fast spin echo (SSFSE) image demonstrating colpocephaly (arrows) in a 26-week gestational age fetus. Panel B is a sagittal T2-weighted SSFSE image demonstrating absence of the corpus callosum. Image reprinted without any changes from Glenn, et al. under the creative common license.

**Figure 5:**
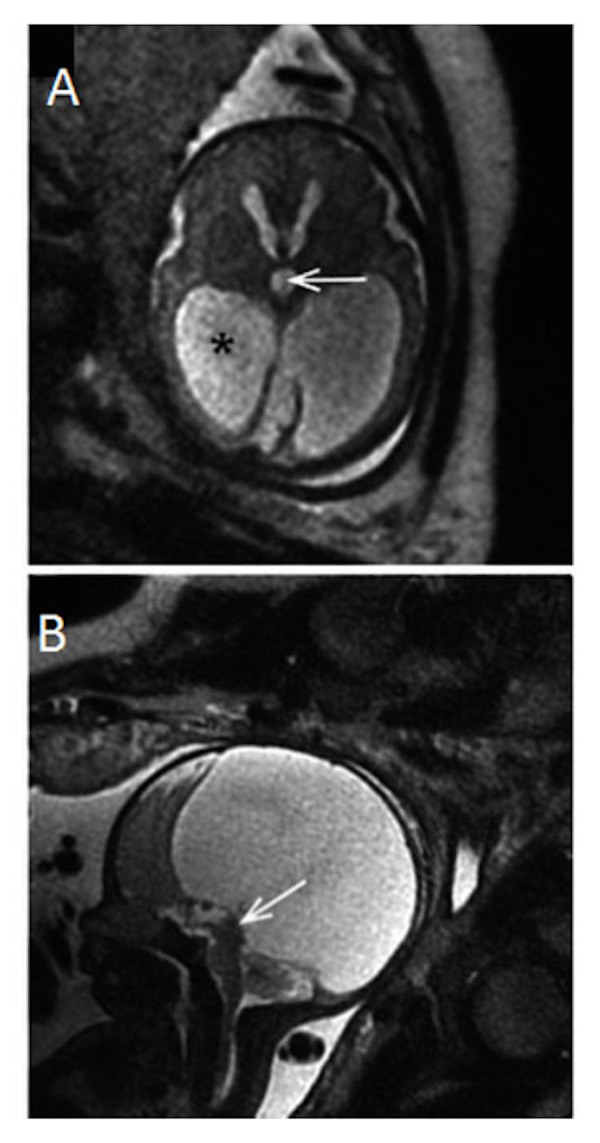
Aqueductal stenosis Panel A is a T2-weighted sagittal image in a 30-week gestational age fetus demonstrating absence of the septum pellucidum, enlargement of the third ventricle (arrow), and ventriculomegaly(*). Panel B is a T2-weighted sagittal image demonstrating lack of a fluid-filled aqueduct of Sylvius (arrow). These findings are consistent with aqueductal stenosis. Image reprinted without changes from Hosseinzadeh, et al. under the creative commons license.

**Figure 6:**
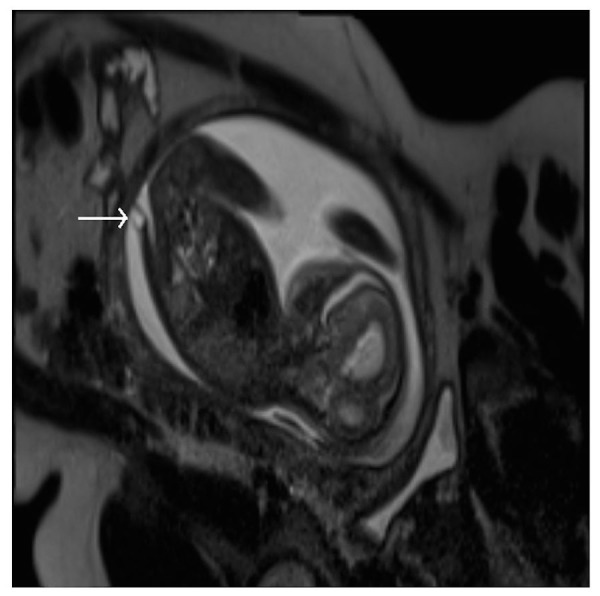
Neural tube defect Sagittal T2-weighted HASTE image demonstrating an open neural tube defect.

**Figure 7:**
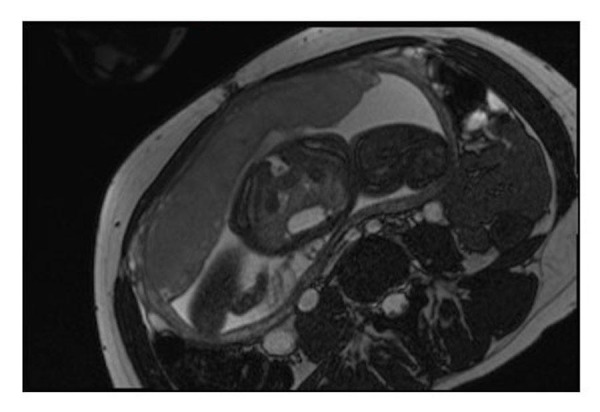
Meningocele An axial T2-weighted image demonstrating a meningocele.

**Figure 8:**
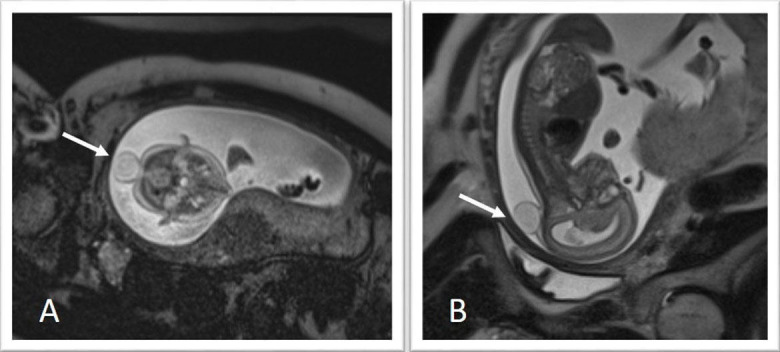
Meningocele Axial (A) and Sagittal (B) T2-weighted HASTE images demonstrate a cystic collection along the dorsal aspect of the occipital region, which is compatible with a meningocele.

Malformations of the central nervous system include asymmetry in cerebral volumes, craniorachischisis, holoprosencephaly (Figure 1), myelomeningocele (Figure 2), spina bifida, Dandy-Walker syndrome (Figure 3), Chiari II malformation (Figure 2), abnormalities of the corpus callosum (Figure 4), aqueductal stenosis (Figure 5), open neural tube defects (Figure 6), spinal meningocele (Figure 7), and occipital meningocele (Figure 8) [31-42].

Fetal MRI for diagnosis of these entities can be performed using a 1.5 Tesla magnet and a multi-coil phased-array torso surface coil. After obtaining localizers, T2-weighted images of the brain should be obtained in three planes utilizing steady-state free precession (SSFP) sequences. For fetuses less than 30 weeks' gestation, an echo time (TE) of 140 should be used, while a TE of 100 should be used for fetuses greater than 30 weeks' gestation. A slice thickness of 3 mm for fetuses less than 24 weeks' gestation and 4 mm for fetuses greater than 24 weeks should be used. Next, a T1-weighted axial image should be obtained, followed by axial diffusion-weighted imaging (DWI) images with apparent diffusion coefficient (ADC) through the brain. Axial images of the brain with echo-planar imaging can be obtained next. T2-weighted images of the body should be obtained in three planes utilizing SSFP sequences to assess the spinal cord. A TE of 8 and slick thickness of 4 mm should be utilized.

For neural tube defects, a slightly modified protocol can be used. The protocol is as described above, including the axial DWI with ADC images. After this, T2-weighted half-Fourier-acquired single-shot turbo spin Echo (HASTE) images should be obtained with slight modifications. Images in the coronal and sagittal planes should be obtained with a TE of 80 and slice thickness of 4 mm. The axial images should be obtained with TE of 140 and slick thickness of 4 mm. T1-weighted gradient-echo (GRE) axial images through the body should be obtained from the cranial portion of the spine to the caudal portion of the defect using 4 mm thick slices. Thereafter, axial images using true fast imaging with steady-state free precession (True-FISP) through the body with 4 mm thick slices should be obtained to complete the evaluation.

### Pulmonary malformations

**Figure 9:**
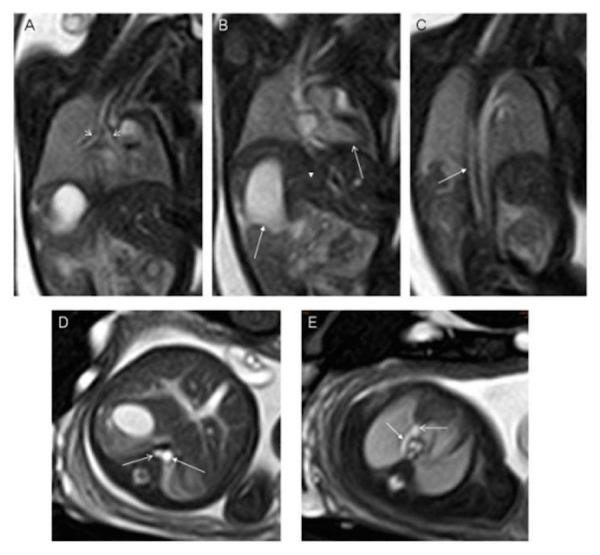
Bronchial isomerism and interruption of the inferior caval vein A balanced turbo field echo imaging of a 28-week gestational age fetus. Panel A is a coronal slice demonstrating bronchial isomerism while panel B demonstrates a right-sided stomach (right-sided arrow), a leftward pointing cardiac apex (left-sided arrow). Panel C demonstrated interruption of the inferior caval vein with azygos continuation (arrow). Panel D is an axial slice demonstrating the prominent azygos vein (arrow) running to the right of the abdominal aorta while panel E demonstrates the azygos vein draining into the superior caval vein. Image reprinted without changes from Dong, et al. under the creative commons license.

**Figure 10:**
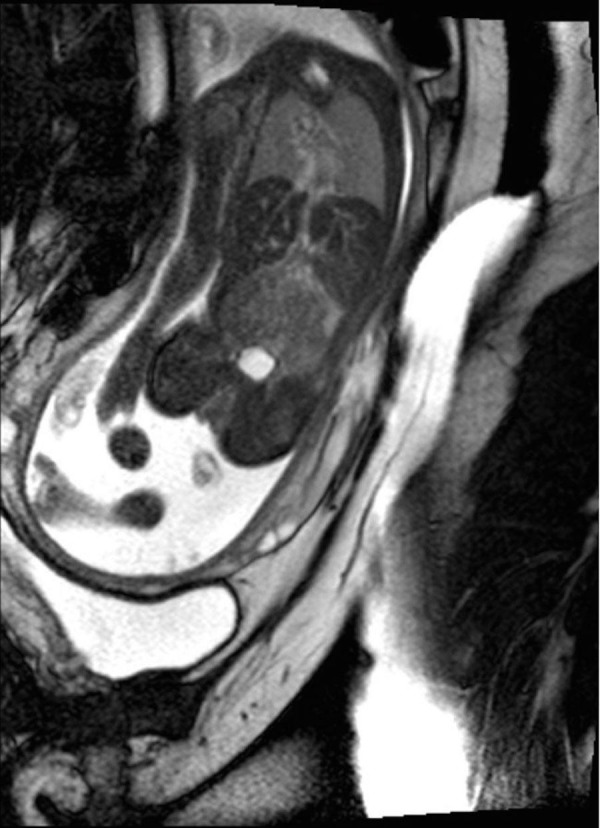
Bronchial isomerism A coronal T1-weighted image demonstrating bronchial isomerism with bronchial angles consistent with right isomerism. There is also a midline liver noted in this slice.

Congenital pulmonary malformations are limited in heterotaxy, but will include isomerism of the bronchi, as revealed by a ratio of bronchial lengths of less than 1.5. Right as opposed to left bronchial isomerism can be determined by assessing the bronchial angles, with angles less than 135 degrees being consistent with left bronchial isomerism, and angles greater than 135 degrees being consistent with right bronchial isomerism (Figures 9-10). The lungs themselves also show isomeric lobation, which is concordant with the bronchial arrangement [25, 43-44].

Assessment of the fetal pulmonary system should begin by three plane localizers, followed by T2-weighted HASTE imaging in three planes. For a fetus with the gestational age of less than 30 weeks, a TE of 140 should be used, with a TE of 100 to be used otherwise with a slice thickness of 4 mm. Next, imaging of the body should be obtained by T2-weighted HASTE images in three planes. Axial images through the body should then be obtained using EPI (bone) to evaluate the vasculature. This should allow for ample visualization of the entire fetus, with a particular focus on the bronchi and lungs.

### Cardiovascular malformations

**Figure 11:**
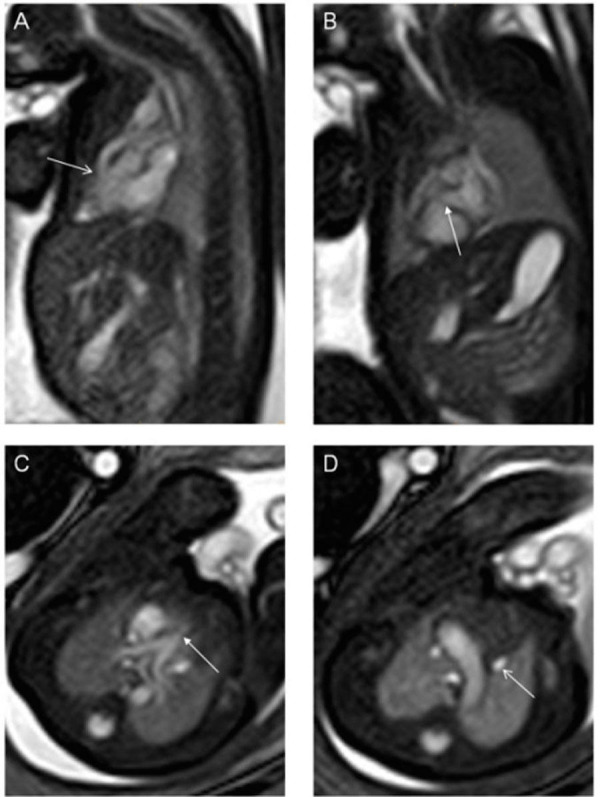
Tetralogy of Fallot Balanced turbo field (bTFE) images of a 28-week gestational age fetus with tetralogy of Fallot. Panel A is a sagittal slice demonstrating obstruction of the right ventricular outflow tract (arrow) while panel B is a coronal slice demonstrating a ventricular septal defect (arrow). Panel C is an axial slice demonstrating valvar pulmonary stenosis (arrow) while Panel D demonstrates a persistent left superior caval vein (arrow). Image reprinted without changes from Dong, et al. under the creative commons license.

**Figure 12:**
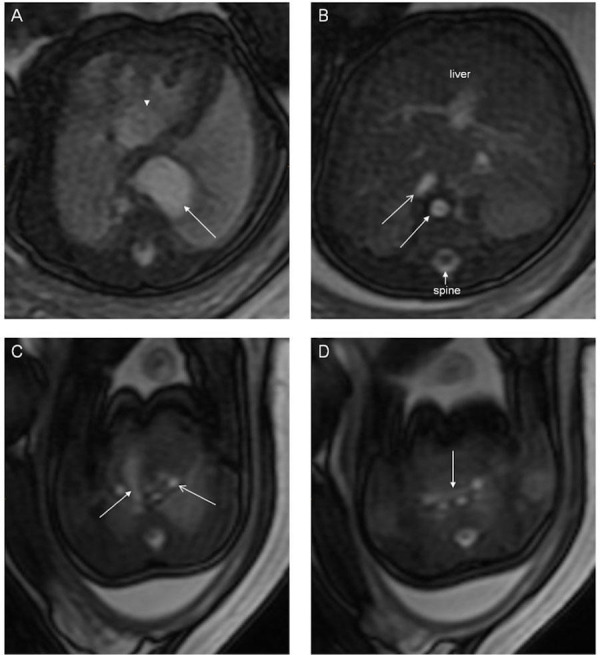
Congenital diaphragmatic hernia Axial FIESTA images of 35-week gestational age fetus with asplenia and congenital diaphragmatic hernia. Panel A is an axial slice demonstrating a left-sided stomach (arrow) that is in the thoracic cavity due to a left-sided congenital diaphragmatic hernia. The heart is pushed into the right chest. Panel B demonstrates an inferior caval vein (open arrow) anterior and rightward to the abdominal aorta (closed arrow). Panel C demonstrates a right aortic arch (closed arrow) and a left sided superior caval vein (open arrow). Panel D demonstrates a right- and left-sided superior caval vein with a bridging vein between the two (arrow). No spleen was identified. Image reprinted without changes from Dong, et al. under the creative commons license.

**Figure 13:**
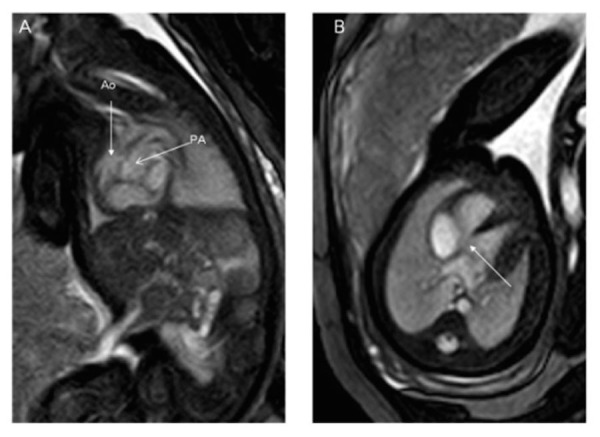
Congenital malformations of the heart Balanced turbo field (bTFE) images of a 34-week gestational age fetus. Panel A demonstrates the aorta arising from the right ventricle as demonstrated by the anterior location of the ventricular mass. The pulmonary artery arises from the left ventricle as demonstrated by the posterior location of the ventricular mass. Panel B is an axial slice demonstrating a ventricular septal defect. Image reprinted without change from Dong, et al. under the creative commons license.

**Figure 14:**
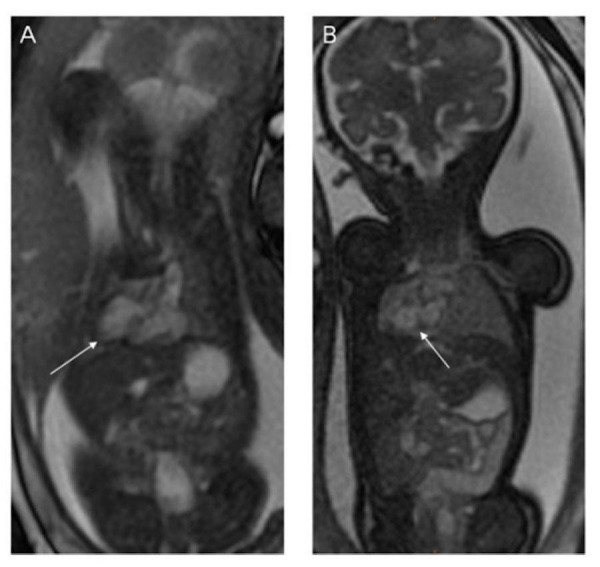
Dextroposition and dextrocardia Fiesta images of a 24 week gestational age fetus. Panel A is a coronal slice demonstrating the heart in the right chest with a leftward pointing apex. There is a left-sided stomach and right-sided liver. Panel B demonstrates the heart in a right chest with a rightward pointing apex. Image reprinted without change from Dong, et al. under the creative commons license.

Cardiovascular malformations in the setting of heterotaxy can vary from simple to complex. While complex cardiovascular malformations can be found with either right or left isomerism, they are more frequently observed with right isomerism. Atrioventricular septal defects and double outlet right ventricle are frequently noted with right isomerism, and ventricular imbalance may often necessitate a univentricular approach to palliation. Other lesions, such as Tetralogy of Fallot (Figure 11), may also be seen. Pulmonary venous connections are, by definition, always anomalous in the setting of right isomerism, even if the pulmonary veins return to the heart. This is because the connections must be anatomically anomalous in the setting of isomeric right atrial appendages. In about half of the cases with right isomerism, nonetheless, the pulmonary veins will drain into an extracardiac confluence. Left isomerism is typically associated with septal defects, coarctation of the aorta, and interruption of the inferior caval vein with azygos continuation (Figures 9, 11). The pulmonary veins in this setting are often connected to the heart in symmetrical fashion. A left-sided superior caval vein may be present with either right or left isomerism (Figures 11-12). In right isomerism, however, the vein will drain to the roof of the left-sided atrium, whereas in left isomerism, it typically drains through the coronary sinus. A right-sided aortic arch may also be present (Figure 12). Discordant ventriculoarterial connections can be found with either variant, but are more common with right isomerism (Figure 13). Left-handed, rather than right-handed, ventricular topology can also be found with either variant. The heart may be in either the left or right chest, while the cardiac apex may point leftward or rightward (Figure 14) [22, 24, 45].

Protocols for cardiac MRI vary considerably as, thus far, experience is limited. After three plane localizers are obtained, it is reasonable to obtain T2-weighted HASTE images in three planes through the body. Next, cine acquisition using True-FISP should be obtained in multiple planes. This can be done by obtaining three orthogonal planes in the thorax, or by using localizers to set up specific image planes corresponding to those of fetal echocardiography. These include transverse views allowing for the equivalent of the four-chamber, five-chamber, pulmonary outflow, and aortic arch views from echocardiography. Sagittal views offer the equivalent of the short axis of the left ventricle, a short axis of the tricuspid and aortic valves, long axis of the arterial duct, and long axis of the aortic arch. Angulated views allow for the provision of the equivalent of long axis views of the left ventricle, and a ductal arch view with both the aortic arch and arterial duct visible. For evaluation of the fetus in its entirety, T2 weighted HASTE images should be obtained in three planes, with a TE of 140 if less than 30 weeks gestational age, and a TE of 100 if greater than 30 weeks gestational age with 4 mm slice thickness. The fetal body should have already been imaged adequately with aforementioned T2-weighted HASTE imaging acquired in three planes.

### Gastrointestinal malformations

**Figure 15:**
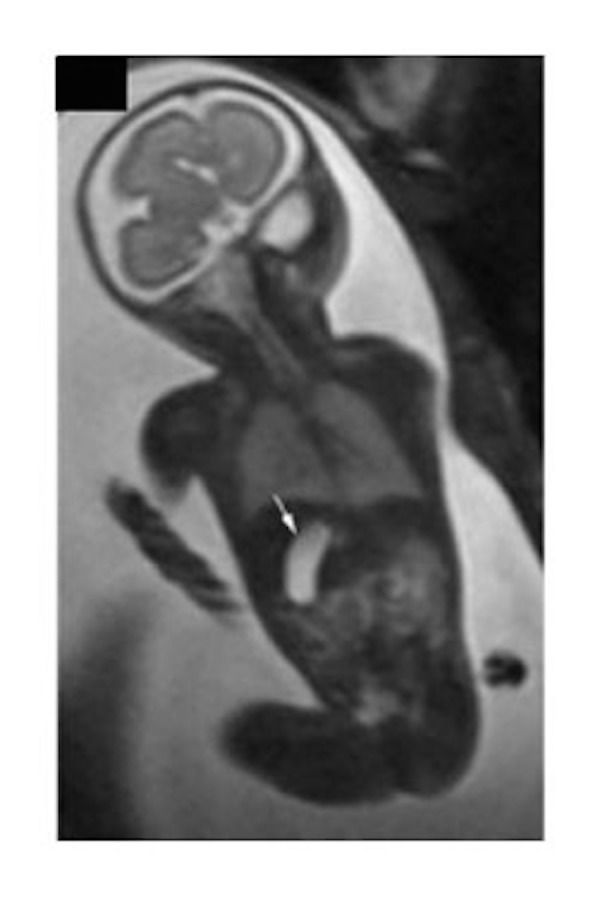
Abnormal abdominal situs T2-weighted HASTE image in the coronal plane demonstrating a right-sided stomach and left-sided liver. Image reprinted without change from Martin, et al. under the creative commons license.

Gastrointestinal malformations are to be anticipated in heterotaxy, with abnormal lateralization of the abdominal organs being the rule. Historically, the position of the abdominal organs has been described in terms of situs solitus, situs inversus, or situs ambiguous. The true value of these terms, however, is limited. Use of “situs ambiguous”, in particular, implies unnecessary uncertainty since it does not provide any account of the location of the different organs. It is best simply to describe the lateralization in terms such as left-sided stomach and right-sided liver for so-called “situs solitus” (Figure 14), right-sided stomach and left-sided liver for “situs inversus” (Figure 15), and right- or left-sided stomach with midline liver for “situs ambiguous” (Figures 9-10). The gallbladder and the pancreas may also lie on the other side of the abdomen from what is expected.

**Figure 16:**
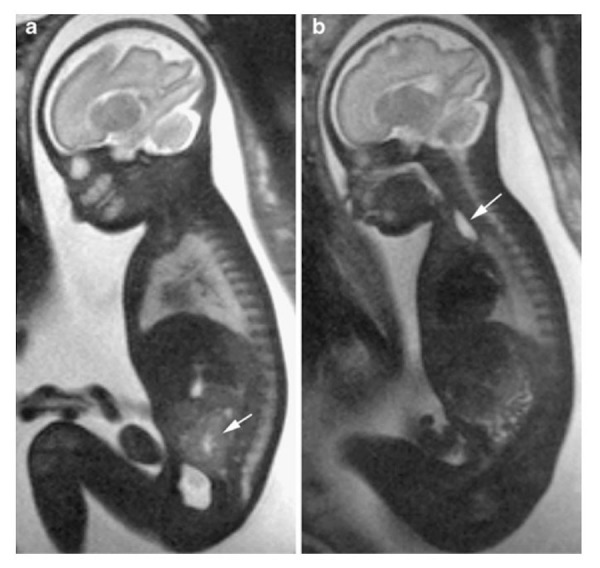
Horseshoe kidney T2-weighted HASTE imaging of a 33-week gestational age fetus. Panel A consists of a sagittal image demonstrating a horseshoe kidney (arrow). Panel B is a sagittal slice also demonstrating a horseshoe kidney and a pouch in the upper esophagus (arrow) consistent with a diagnosis of tracheoesophageal fistula. Image reprinted without change from Martin, et al. under the creative commons license.

**Figure 17:**
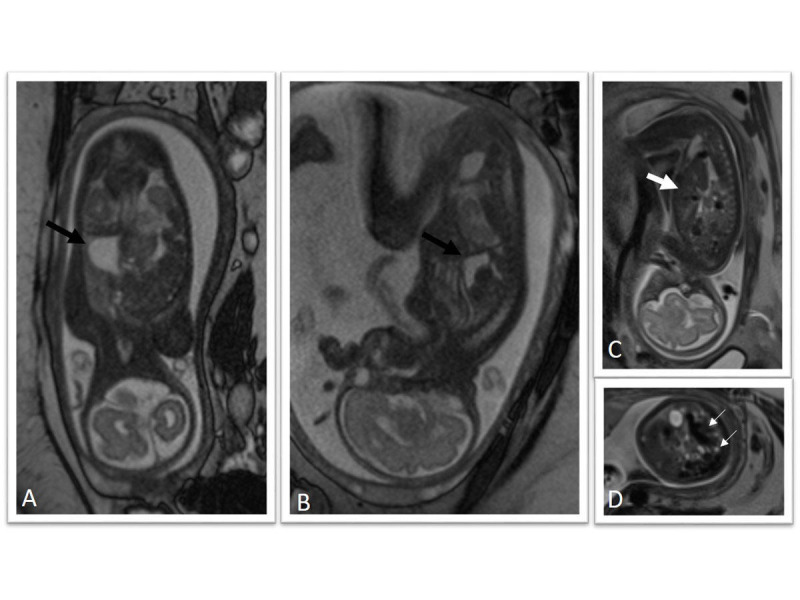
Congenital diaphragmatic hernia Coronal (A), sagittal (B and C), and axial T2-weighted HASTE images demonstrate a large left-sided diaphragmatic hernia containing stomach (black arrow), left hepatic lobe (thick white arrow), and loops of bowel (small white arrows).

**Figure 18:**
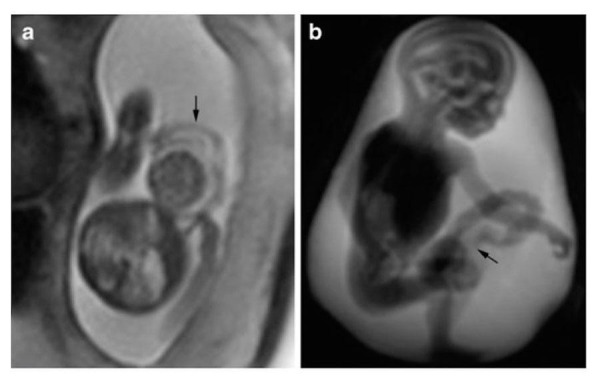
Omphalocele T2-weighted HASTE imaging of a 21-week gestational age fetus. Panel A is an axial image demonstrating intestinal loops lined with peritoneal membranes that are outside of the abdomen. Panel B is a sagittal slice demonstrating bowel loops outside of the abdomen as well as insertion of the umbilical cord. Image reprinted without change from Martin, et al. under the creative commons license.

Apart from abnormal lateralization of the major organs, other gastrointestinal malformations are to be anticipated. While not purely gastrointestinal malformations, tracheoesophageal fistulas (Figure 16) and congenital diaphragmatic hernias (Figure 17) can be associated with heterotaxy [14, 37, 46-48]. Perhaps the most frequent gastrointestinal manifestation, nonetheless, is malrotation [49]. Routine screening for malrotation in the setting of heterotaxy is currently under debate, since screening studies in asymptomatic infants likely offer no benefit [50]. Prophylactic Ladd’s procedures carried out in asymptomatic patients may be harmful, particularly in patients who have had cardiac palliation with a shunt, since the procedure increases the risk of shunt thrombosis [51]. Omphalocele (Figure 18) has also been noted in those with heterotaxy and is always associated with a degree of malrotation [14, 48, 52-53]. Biliary atresia, duodenal atresia, agenesis of the dorsal pancreas, and anal atresia are also found [54-58]. Heterotaxy, furthermore, is known to carry a higher risk of portosystemic shunts, known as Abernethy malformations [59-61].

When imaging the fetus with a focus on the body, localizers should be obtained in three planes to determine the fetal lie. Next, T2-weighted HASTE images of the brain should be obtained in three planes. Once again, a TE of 140 should be used for gestational age of less than 30 weeks and 100 if greater than 30 weeks with a slice thickness of 4 mm. Next, T2-weighted HASTE images of the body should be obtained in three planes with a TE of 80 and slice thickness of 4 mm. SSFP images should then be obtained in three planes using a flip angle of 110 and slice thickness of 6 mm. This should be followed by T1-weighted coronal images of the body to assess for anorectal malformations. If there is a particular concern for a tracheoesophageal fistula, then T1-weighted sagittal images of the body should be obtained.

### Immunologic malformations

Abnormalities of the spleen are to be expected in those with heterotaxy. While not absolute rules, multiple spleens are more commonly noted in those with left isomerism, with an absence of the spleen typically found in those with right isomerism. Some fetuses, nonetheless, will have a normally-sized solitary spleen, which can either be right- or left-sided [23-24]. While discussion of visceral function is beyond our current scope, splenic dysfunction can be noted, even in the presence of a normally located solitary spleen, and in those with multiple spleens [29]. The spleen can be assessed using the fetal body protocol as outlined for assessment of gastrointestinal malformations. Imaging the spleen, however, may be difficult and may not be reliable in the fetus.

### Genitourinary malformations

**Figure 19:**
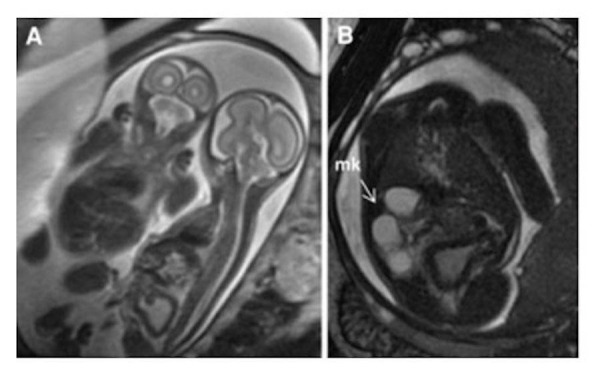
Multicystic kidney T2-weighted HASTE imaging of monochorionic, monoamniotic twins. Panel A demonstrates the affected twin is on the right with a thickened and distended bladder. Panel B is an axial slice demonstrating a multicystic kidney (arrow). Reprinted without change from Bischoff, et al. under the creative commons license

Heterotaxy is associated with malformations of the genitourinary system, including horseshoe kidney (Figure 16), ectopic ureters, ureteral duplication, cystic kidneys (Figures 1, 19), solitary kidney, and cloacal duplication [58, 62-63]. These malformations may be assessed using the fetal body protocol outlined above, although all stacks should be extended to include the fetal pelvis.

### Other malformations

There are few other malformations that do not fall into one of the other categories above. Cleft lip and cleft palate, for example, do not lend themselves to any of the previous categories, but are known to exist in heterotaxy. Imaging of the cleft lip or cleft palate can be done with any of the above protocols, with attention to ensure that brain images are extended to include the entirety of the fetal head.

## Conclusions

As the indications for fetal MRI increase, so does the need to recognize underlying syndromes or clinically recognized constellations of symptoms, such as is known to exist in so-called visceral heterotaxy. Associated findings should prompt more detailed assessment of other systems than those of the original interest. The findings will then better facilitate appropriate counseling. 
